# Toward optimal implementation of cancer prevention and control programs in public health: a study protocol on mis-implementation

**DOI:** 10.1186/s13012-018-0742-9

**Published:** 2018-03-23

**Authors:** Margaret Padek, Peg Allen, Paul C. Erwin, Melissa Franco, Ross A. Hammond, Benjamin Heuberger, Matt Kasman, Doug A. Luke, Stephanie Mazzucca, Sarah Moreland-Russell, Ross C. Brownson

**Affiliations:** 1 Prevention Research Center in St. Louis, Brown School at Washington University in St. Louis, 1 Brookings Drive, Campus Box 1196, St. Louis, MO 63130 USA; 2 Department of Public Health, University of Tennessee, Knoxville, TN USA; 3 Center on Social Dynamics and Policy, The Brookings Institution, Washington DC, USA; 4 Center for Public Health System Science, Brown School at Washington University in St Louis, St. Louis, MO USA; 5 Department of Surgery (Division of Public Health Sciences) and Alvin J. Siteman Cancer Center, Washington University School of Medicine, Washington University in St. Louis, St. Louis, USA

**Keywords:** Mis-implementation, Cancer control, Agent-based models

## Abstract

**Background:**

Much of the cancer burden in the USA is preventable, through application of existing knowledge. State-level funders and public health practitioners are in ideal positions to affect programs and policies related to cancer control. Mis-implementation refers to ending effective programs and policies prematurely or continuing ineffective ones. Greater attention to mis-implementation should lead to use of effective interventions and more efficient expenditure of resources, which in the long term, will lead to more positive cancer outcomes.

**Methods:**

This is a three-phase study that takes a comprehensive approach, leading to the elucidation of tactics for addressing mis-implementation. Phase 1: We assess the extent to which mis-implementation is occurring among state cancer control programs in public health. This initial phase will involve a survey of 800 practitioners representing all states. The programs represented will span the full continuum of cancer control, from primary prevention to survivorship. Phase 2: Using data from phase 1 to identify organizations in which mis-implementation is particularly high or low, the team will conduct eight comparative case studies to get a richer understanding of mis-implementation and to understand contextual differences. These case studies will highlight lessons learned about mis-implementation and identify hypothesized drivers. Phase 3: Agent-based modeling will be used to identify dynamic interactions between individual capacity, organizational capacity, use of evidence, funding, and external factors driving mis-implementation. The team will then translate and disseminate findings from phases 1 to 3 to practitioners and practice-related stakeholders to support the reduction of mis-implementation.

**Discussion:**

This study is innovative and significant because it will (1) be the first to refine and further develop reliable and valid measures of mis-implementation of public health programs; (2) bring together a strong, transdisciplinary team with significant expertise in practice-based research; (3) use agent-based modeling to address cancer control implementation; and (4) use a participatory, evidence-based, stakeholder-driven approach that will identify key leverage points for addressing mis-implementation among state public health programs. This research is expected to provide replicable computational simulation models that can identify leverage points and public health system dynamics to reduce mis-implementation in cancer control and may be of interest to other health areas.

## Background

Cancer continues to be the second most common cause of death in the USA [1, 2]; however, much of this burden is preventable through evidence-based interventions [3]. Substantial potential for cancer control exists at the state level [4, 5] in which all states retain enormous authority to protect the public’s health [6]. States shoulder their broad public health responsibilities through work carried out by state and local health agencies. Over $1.1 billion annually is expended on state cancer control programs^1^ (i.e., primary and secondary prevention) [7, 8], which is significantly higher than any other area of chronic disease prevention and control. However, cancer control covers a broad spectrum of programs, and funding can be limited in areas and population groups with high cancer burdens [9]. With the limited resources available to state-level programs, the need to utilize the best available evidence to implement and sustain these programs is key to the efficiency of cancer control at the state level [10].

Evidence-based approaches to cancer control can significantly reduce the burden of cancer [10–13]. This approach begins with an estimate of the preventable burden. Depending on the methods, between one third and one half of deaths due to cancer can be preventable [3, 14, 15]. Large-scale efforts such as Cancer Control P.L.A.N.E.T. and the *Community Guide* have now placed a wide array of evidence-based interventions in the hands of cancer control practitioners [13, 16, 17]. Despite those efforts, a set of agency-level structures and processes (e.g., leadership, organizational climate and culture, access to research information) needs to be present for evidence-based decision-making (EBDM) to grow and thrive [10, 18–20]. While efforts are building to make sure practitioners have access to and the capacity for EBDM [21], the need for the exploration of mis-implementation of these programs in public health is growing.

### Importance and potential impact of mis-implementation

The scientific literature has begun to highlight the importance of considering de-implementation in health care and public health [22, 23]. While de-implementation looks at the retraction of unnecessary or overused care [23, 24], it does not quite fully examine the processes that sustain non-evidence-based programs and the de-implementation of programs that are, in fact, evidence-based. An example of the discontinuation of an evidence-based program is notable with the VERB campaign in the USA that demonstrated effectiveness in increasing physical activity of children but was then discontinued [25, 26]. On the other end of the mis-implementation spectrum is the continuation of non-evidence-based programs such as the continuation of the DARE (Drug Abuse Resistance Education) program despite many evaluations have demonstrated its limited effectiveness [27, 28]. That is why researchers have come to define mis-implementation as the process where effective interventions are ended or ineffective interventions are continued in health settings (i.e., EBDM is not occurring) [22, 24]. Most of the current literature focuses on the overuse and underuse of clinical interventions and the cultural and organizational shifts needed toward the acceptance of de-adoption within medicine [29]. Currently, over 150 commonly used medical practices have been deemed ineffective or unsafe [23]. Despite this discovery within the medical realm, there is still sparse literature on mis-implementation in the field of public health or cancer control.

It is already known that there are a number of cancer control programs that continue without a firm basis in scientific evidence [12]. Hannon and colleagues reported that less than half of cancer control planners had ever used evidence-based resources [12]. Previous studies have suggested that between 58 and 62% of public health programs are evidence-based [30, 31]. Even among programs that are evidence-based, 37% of chronic disease prevention staff in state health departments reported programs are often or always discontinued when they should continue [22].

### Factors likely to affect mis-implementation

In delivery of mental health services, Massatti and colleagues made several key points regarding mis-implementation: (1) the right mix of contextual factors (e.g., organizational support) is needed for the continuation of effective programs in real-world settings; (2) there is a significant cost burden of the programs to the agency; and (3) understanding the nuances of early adopters promotes efficient dissemination of effective interventions [32]. Management support for EBDM in public health agencies are associated with improved public health performance [18, 33], but little is known about the processes and factors that affect mis-implementation specifically. Pilot data indicate that organizational supports for EBDM may be protective against mis-implementation (e.g., leadership support for EBDM, having a work unit with the necessary EBDM skills) [22]. In addition, engaging a diverse set of partners may also lower the likelihood of mis-implementation.

### The utility of agent-based modeling for studying public health practice

Agent-based modeling (ABM) is a powerful tool being used to inform practice and policy decisions in numerous health-related fields [34]. ABM is a type of computational simulation modeling in which individual agents—who may be people, organizations, or other entities—are defined according to mathematical rules and interact with one another and with their environment over time [35]. ABM is a useful tool to observe the dynamic and interdependent relationships between heterogeneous agents within a complex system and how system-level behavior and outcomes evolve over time from the interaction between these individual agents (emergent behavior) [35–37].

Agent-based models have a strong track record in social and biological sciences and have been widely used to study infectious disease control [38, 39] and health care delivery [40, 41]. More recently, ABM has begun to be applied to chronic disease control, ranging from the study of etiology, to intervention design, to policy implementation [35, 40, 42–44]. In topics specific to cancer prevention, ABMs are now being used to show how individuals respond to tobacco control policies [45] and to better understand the community-level, contextual factors involved in implementing childhood obesity interventions [43]. Some of the advantages of employing ABMs include that they can (1) model bi-directional and non-linear interactions between individuals, organizations, and external contextual factors [46]; (2) describe dynamic decision-making processes [47]; (3) simulate adaptation, counterfactuals, and relational structures such as networks [34, 42]; (4) consider and capture extensive heterogeneity across different entities or populations [48]; and (5) act as “policy laboratories” for researchers when real-world experimentation is not feasible or is too costly [48]. ABM has the potential to pinpoint both the factors within state health departments that have the greatest effect on the mis-implementation of cancer control programs and the leverage points that may be good targets to improve successful implementation.

Early on, ABMs primarily relied on simple heuristic rules as models of human behavior and were generally limited in their ability to predict behaviors of larger populations and complex interactions. In recent years, however, ABMs have provided (1) more refined representations of behavior and decision-making [49, 50], (2) increasingly sophisticated representations of relational/environmental structures such as geography and networks [34, 42], and (3) greater focus on “co-evolution” across levels of scale across settings, including organizational dynamics as in political science [34, 47].

## Methods/design

**Fig. 1:**
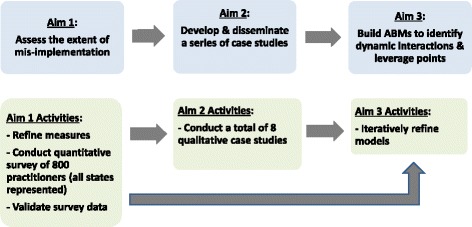
Study schema

This is a multi-part, observational study funded by the National Cancer Institute that will examine the factors that affect mis-implementation of cancer control programs. ABM will be used here to understand influences of individual, organizational, and external factors on program implementation, decision-making, and the behaviors of professionals within state health departments. See Fig. 1 for a visual of the study schema. The project is outlined in the following three phases: (1) assessing mis-implementation, (2) conducting comparative case studies, and (3) agent-based modeling. The study design has been approved by the Washington University Institutional Review Board.

### Phase 1, assessing mis-implementation (Aim 1)

The measures to assess the scope and patterns of the mis-implementation problem are vastly under-developed. There has been limited pilot work in this area [22]; therefore, phase 1 will focus on the refinement of measures and assessment of the patterns of mis-implementation in cancer control in the USA. The project begins by refining and pilot testing measures to assess mis-implementation within state health departments. The foundation of these measures comes from a pilot survey previously completed by members of the team, the Mis-Implementation Survey for Cancer Control [22, 51–58]. The project team has engaged a group of public health practitioners who will serve as an advisory group throughout the duration of the project and will help inform the development of the measures.

**Fig. 2:**
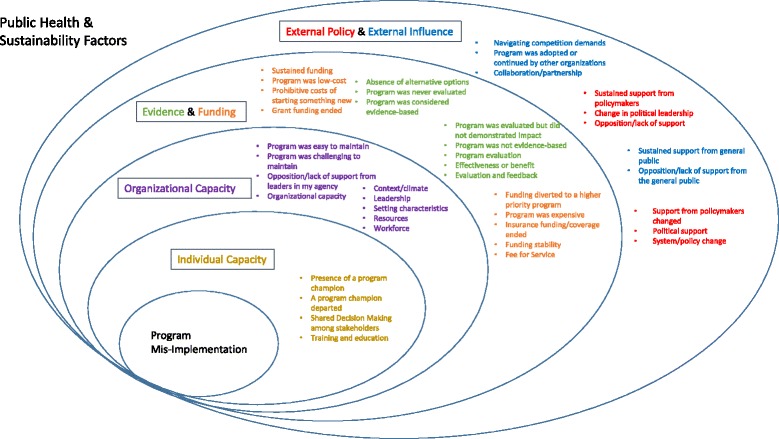
Conceptual framework for mis-implementation

The first step in exploring mis-implementation is to build the self-report survey instrument. To do so, the team will search for existing instruments using formal queries of the published literature. The team will search public health, sustainability, and clinical and agent-based modeling literature. Search terms include Evidence-based decision making AND De-Implement* AND Measures; sustain*; de-implement* AND Measures; de-adopt* OR de-implement*; de-implement AND Health AND instrument; Organization* Capacity AND Measures. Candidate instruments will also be provided by the research team, lead authors of collected literature, and bibliography reviews [59]. Identified measures will be cataloged, and the authors will be queried for the related instruments. Relevant instruments will be summarized into evidence tables, highlighting core constructs, audience, and measurement properties. See Fig. 2 for the conceptual framework that will guide the survey development.

Using the evidence tables, a draft instrument will be developed. It is likely to cover seven main domains: (1) biographical information; (2) frequency of mis-implementation; (3) reasons for mis-implementation; (4) barriers in overcoming mis-implementation; (5) specific programs being mis-implemented; (6) use of management supports for EBDM; and (7) ratings on current level of individual skills essential for implementing evidence-based interventions.

New measures will undergo expert review for content validity, relying on the advisory group of state health department practitioners. Before the instrument goes into the field, a series of individual interviews will be completed for cognitive response testing of newly developed items. Cognitive response testing is routinely used in refining questionnaires to improve the quality of data collection [60–62]. Cognitive response testing will be used to determine: (1) *question comprehension* (what does the respondent think the question is asking?); (2) *information retrieval* (what information does the respondent need to recall from memory in order to answer the question?); and (3) *decision processing* (how does the respondent choose their answer?).

Once cognitive testing is completed, additional edits will be made to the survey, and a test-retest will be employed to assess reliability of the instrument. The team intends to recruit around 100 practitioners, via the advisory group to complete the survey and then complete the survey again, 2 weeks after initial administration. Appropriate statistics will be calculated for each type of question to assess the reliability between the two test time points [63, 64].

#### Study participants

Study participants will include cancer control public health practitioners, which include those individuals who direct and implement population-based intervention programs in state health departments. These practitioners may be directly involved in program delivery, may set priorities, or allocate resources for programs related to cancer risk factors. The target audience will be inter-disciplinary; that is, they will be drawn from diverse backgrounds including health educators, epidemiologists, and evaluators. Examples of the individuals in the target audience include (1) the director of a Centers for Disease Control and Prevention (CDC)-funded comprehensive cancer control program for the state; (2) the director of a state program addressing primary prevention of cancer (tobacco, inactivity, diet, sun protection); (3) the director of state programs promoting early detection of breast, cervical, and colorectal cancers among underserved populations; or (4) state health department epidemiologists, evaluators, policy officers, and health educators supporting cancer control programs.

Participants will be randomly drawn from the 3000-person membership of National Association of Chronic Disease Directors (NACDD) and program manager lists from key CDC-supported programs in cancer and cancer risk factors. The team has an established partnership with NACDD and has worked extensively with them on previous projects [30, 54–57, 65–68]. In phase 1, the team will recruit 1040 individuals for a final sample size of 800. The team anticipates a 60% response rate based on evidence that supports a series of emails, follow-up phone calls, and endorsements from NACDD leadership and officials in the states with enrolled participants [51, 52, 54, 56, 69–74]. Similar to successful approaches in previous studies [56, 74], data will be collected using an online survey (Qualtrics software [75]) that will be delivered via email. The survey will remain open for a 2-month time period with four email reminders and two rounds of phone calls to bolster response rates [72]. All respondents will be offered a gift card incentive.

#### Analyzing the survey data

Survey data will be analyzed in three ways. First, descriptive statistics (e.g., frequencies, central tendencies, and variabilities) and diagnostic plots (e.g., stem and leaf plots, q-q plots) will be completed on all variables. Data will be examined for outliers and tested as appropriate for normality, linearity, and homoscedasticity. Appropriate corrective strategies will be used if problems are identified. Bivariate and multivariate analyses will rely on data at the single time point of the phase 1 survey. These preliminary analyses are necessary to ensure high-quality data and to test assumptions of the proposed models. The team will also compare demographic and regional variations between respondents and non-respondents to assess potential response bias.

Next, the measurement properties of the instrument will be comprehensively assessed. To do so, the team will conduct confirmatory factor analysis within an explanatory framework using structural equation models [76, 77]. Using factor analysis seeks to reduce the anticipated large number of items in the survey tool to a smaller number of underlying latent variables while examining construct validity of the measures [78]. The initial domains to be used in factor analysis are shown in Fig. 2.

Finally, multivariate analyses will use general linear or logistic mixed-effects modeling. A mixed-effects approach allows the team to include continuous predictors, both fixed and random categorical effects, and allows us to model the variability due to the nested design (individuals nested in health departments). The mixed-effects model will assess effects of multiple predictors on two key dependent variables (*Y*_*ij*_) (ending programs that should continue, continuing programs that should end). The team will be able to assess covariate effects of numerous variables such as gender, educational background, and time in current job position. Here is a sample model:

$$ {Y}_{ij}={\beta}_{0j}+{\beta}_1{P}_{ij}+{\beta}_2{R}_{ij}+{\beta}_3{S}_{ij}+{\beta}_4{(PR)}_{ij}+{\mu}_j+{\varepsilon}_{ij} $$

where *P*_*ij*_ and *S*_*ij*_ are the fixed effects for program type and state population size, *R*_*ij*_ is a fixed effect for reason for mis-implementation, (*PR*)_*ij*_ is the program by reason interaction, and *μ*_*j*_ and *ε*_*ij*_ are the variance components at the state and individual levels, respectively. The mixed-effects modeling allows for random effects at the state (*μ*_*j*_) level. A mixed-effects model will examine state-level variability and account for the nested design of the study [79].

#### Sample size calculations

For the factor analysis, given a minimum of four items per factor and expected factor loading of 0.6 or higher, the survey will need a sample of 400 [80]. For reliability testing, for statistically significant (*p* < 0.05) kappa values of 0.50 and 0.70, the sample size requirements are 50 and 25 pairs, respectively, in each of the two groups. To estimate an intraclass correlation coefficient of 0.90 or above (power 0.80, *p* < 0.05), 45 pairs are required in each subgroup. Sample size estimates are based on Dunn’s recommendations [81]. Therefore, a sample of 100 for reliability testing will provide high power.

For population-level estimates of mis-implementation and multivariate modeling, sample sizes are based on a power ≥ 90% with two-sided *α* = 5% [22]. To estimate a prevalence of mis-implementation of 37% (± 3%), a sample of 750 is needed. To compare rates of mis-implementation by program area (e.g., cancer screening estimated at 19% versus primary prevention of cancer estimated at 29%), a sample size of 800 is required at power > 0.90 and *p* < 0.05.

### Phase 2, comparative case studies (Aim 2)

Often, the key issue in understanding the translation of research to practice is not the evidence-based intervention itself, but rather the EBDM *processes* by which knowledge is transferred (i.e., *contextual evidence* as described by Rychetnik et al. [82] and Brownson et al. [10]) [83]. Building on data collected in phase 1, the goal of phase 2 is to better understand the context for mis-implementation via case studies, which will involve key informant interviews. These interviews will involve sites that are successful or less than successful in addressing mis-implementation. The purpose of key informant interviews is to collect information from a range of people who have first-hand knowledge of an issue within a specific organization or community.

#### Sampling, recruitment, and interview domains

The team will utilize purposive sampling to select participants [84]. Based on phase 1 data, eight states will be selected—that is, four states where mis-implementation is high and four where mis-implementation is low. Participants will be state health department practitioners who work in the identified states. By examining extreme cases, the study can maximize the likelihood that the qualitative approach will provide deeper understanding of mis-implementation, building on Aim 1 activities. While Aim 1 will determine the extent (the “how much”) and some underlying reasons (the “why”), Aim 2 will give a deeper understanding of the “why” and “how” of mis-implementation. The interviews will focus on several major areas (organized around domains in Fig. 2): (1) inputs related to mis-implementation, (2) factors affecting mis-implementation (individual, organizational, external), and (3) methods for reducing mis-implementation.

#### Data collection

Study staff will make initial contact via email and by telephone to invite identified participants to the study and arrange an appointment for the telephone interview. Participants will receive consent information in accordance with the Washington University Institutional Review Board standards. The team anticipates approximately 12 interviews from each of the eight state health departments (a total of 96 interviews) and will conduct interviews until the team reaches thematic saturation [85]. Participants will be offered a gift card incentive.

#### Data coding and analysis

Digital audio recordings of the interviews will be transcribed verbatim. Two project team members will analyze each of these transcripts via consensus coding. After reviewing the research questions [86, 87], the team members will read five of the transcripts using a first draft of a codebook. Each coder will be asked to systematically review the data and organize each of the statements into categories that summarize the concept or meaning articulated [88]. Once the first five transcripts are coded, they will be discussed in detail to ensure the accuracy of the codebook and inter-coder consistency. The codebook will be edited as needed prior to coding the remainder of the transcripts. All transcripts will be analyzed using NVIVO.11 [89]. After refinement of the codebook, each transcript will be coded independently by two team members. The two team members will then review non-overlapping coding in the text blocks and reach agreement on text blocking and coding. Themes from the coded transcripts will be summarized and highlighted with exemplary quotes from participants. Data analysis may also include quantification or some other form of data aggregation. The study team will use the interview guide questions to establish major categories (e.g., individual factors, organizational factors). All information that does not fit into these categories will be placed in an “other” category and then analyzed for new themes. Comparisons will be made to identify key differences in thematic issues between those in high and low mis-implementation settings. After initial analyses by research team members, one focus group will be conducted remotely or in-person with state health department staff in each of the eight states to get input on other interim theme summaries.

### Phase 3, agent-based modeling

**Fig. 3:**
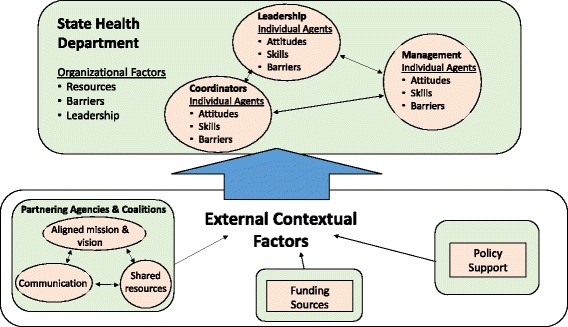
Agent-based model flowchart

Agent-based modeling will allow the study team to explore how mis-implementation emerges through interactions between individuals who are situated within public health organizations. The general approach will be to model a hypothetical public health program environment, populated by individuals (agents) who are programmatic decision-makers or influencers within state health departments. The key behavior of interest in the simulation models will be the decision to continue or discontinue strategies within cancer prevention and control programs in a state health department. The resulting model will have the ability to simulate both overall trajectory of decisions being made as well as the patterns of interacting factors that emerge in these types of decision-making processes [34]. The models will consider individual characteristics (from the survey) and organizational characteristics (from the survey and archival data) across an agency hierarchy as well as influences from contextual, external forces that affect the state health department environments (e.g., policy changes, strengths of partnerships) and their potential effects on mis-implementation (Fig. 3 and Table 1).

**Table 1:** Sample agents and contextual factors

Domain	Model-relevant construct	Sample elements	Sources
Agent (individual)	Attitudes	- Appeal of evidence-based practices - Openness to innovation	Aarons et al. [97]
	Skills	- Use economic evaluation in the decision-making process - Adapt programs for different communities and settings	Gibbert et al. [31] Jacob et al. [56]
	Barriers	- Lack of knowledge of EBDM - General resistance to changing old practices	Maylahn et al. [98] Jacobs et al. [57]
Agent (organizational)	Resources	- Program funding - Expertise of available staff	Erwin et al. [99] Brownson et al. [52]
	Leadership/management support	- Agency leadership values EBDM - Management practices of direct supervisor	Brownson et al. [18] Jacob et al. [56]
	Barriers	- Lack of incentives in the agency for EBDM - Organizational culture does not support EBDM	Jacobs et al. [57] Gibbert et al. [31]
Contextual (external)	Partnerships	- Maintains a diverse set of partners - Compatible processes between partners	Brownson et al. [18] Massatti et al. [32]
	Policy support	- Supportive state legislature - Supportive governor	Brownson et al. [52]

#### Defining the agents and contextual factors

Agents in an ABM are generally defined by individual characteristics (properties), behavior rules that govern choices or actions (possibly dependent on both the agent’s own state and that of the environment) [36, 37], and a social environment that characterizes relationships between agents. In this case, both individual agents (the public health professionals working in cancer prevention and control within state health departments) and organizational agents (the state health departments in which decisions are being made) will be influenced by elements outside the state health department, as well as by interactions with each other.

The complexity of the systems in which the organizational and external factors operate and influence public health practitioner decision-making is such that ABM will be able to provide greater insight than traditional experimental design or epidemiological and econometric analytic tools which require assumptions about homogeneity, linearity, etc. that are not appropriate for complex organizational systems [36, 90]. By developing a model that is informed by survey and case study data, we will be able to help explain how mis-implementation arises from decisions made by individuals within specific organizational and external contexts. Additionally, ABM can provide insight into potential counterfactuals and implications these may have for intervention designs and targeting (e.g., if a particular organizational climate had been present, mis-implementation would not have occurred). The potential benefits of using an ABM approach include anticipating both the individual impact of modifiable influences on decision-making and the organizational impacts of mis-implementation in varying conditions [43].

In our planned initial model design, individual-level agents will work within a health department, making periodic choices about whether to continue or discontinue specific intervention strategies within cancer prevention and control programs. The team will explore the impact that the underlying factors of state health departments have on the patterns of mis-implementation. The team has developed the initial set of individual and organizational agent constructs and external influences from pilot research in mis-implementation and in evidence-based decision-making [18, 21, 22, 31, 56, 57, 74] and draws on previous literature from systems science on organizational dynamics both within health systems and other fields [91–96]. Findings from phases 1 and 2 will allow the team to refine the core list of agent factors and also estimate the relative importance of different agent behaviors and attributes using a “bottom-up” approach (i.e., real-world data collected from individuals and organizations). Several of the agent factors listed in Table 1 have predicted mis-implementation in pilot research.

##### Individual

Individual-level agents in the initial models will represent the staff members who work within state health departments. These individuals, who exist in a hierarchy within a state health department, include leaders, managers, and coordinators (Fig. 3). The potential characteristics for modeling include their attitudes (e.g., openness to innovation) [97], skills and knowledge (e.g., ability to use economic evaluation) [31, 56], and barriers (e.g., lack of knowledge of EBDM processes) [57, 98], specifically those that may contribute to mis-implementation. Individual agents will also have social connections with other agents that affect how information flows through the organization and how organization-level decisions about implementation are made.

##### Organizational

The second set of planned agents are organizations representing state health departments. These organizations have characteristics that influence mis-implementation [22]. Several organizational agent characteristics likely to affect mis-implementation are management supports [18, 56], resources [52, 99], and organizational barriers [31, 57].

##### Contextual (external) factors

In addition, the team plans to model external contextual factors. While these are not directly present within health departments, context can have a significant influence on whether program strategies are continued or discontinued. The initial set of external factors has been drawn from pilot research and previous conceptual frameworks and includes variables such as a diverse, multi-disciplinary set of partners with EBDM skills [18, 32], funding climate, policy inputs, and political support [52]. The exploration of external factors within the models will allow the team to observe how agents adapt to different contexts—including through departmental and organizational communication channels—and how adaptation affects outcomes related to mis-implementation [34].

#### Developing the computational simulation

The development of the ABM will follow established computational modeling best practices [100]. The modeling process will have four key steps:

##### Step 1: model design and internal consistency testing

Design of the models begins with identification of key concepts and structures from the literature and pilot studies, as well as phases 1 and 2 and their operationalization into appropriate model constructs. Table 1 is a start from the pilot data and will be refined from what the team learns in phases 1 and 2. The models are then implemented in computational architecture, with each piece undergoing testing to ensure appropriate representation of concepts and implementation. Revisions of initial models’ implementation are undertaken as needed based on partial model testing and consultation with experts.

##### Step 2: test for explanatory insights

Once the initial models are complete, the generative explanatory power of the models to reproduce real-world observations about mis-implementation can be tested under a variety of different conditions. The testing procedure will have two parts. In part one, the ABM will be used to examine mis-implementation related to ending programs that should continue. In part two, the team will examine mis-implementation related to continuing programs that should end. For each type of mis-implementation, the team will focus on the ability of the models to reproduce “stylized facts” about mis-implementation obtained from pilot studies, activities in phases 1 and 2, and from the advisory group. These “stylized facts” are used to calibrate the models and include variables such as how skilled individuals are in EBDM and how strongly the organizational climate and culture supports EBDM. The engagement of the advisory group will be essential in this step.

##### Step 3: sensitivity analyses

Systematic exploration of model behavior will be undertaken as key parameters and assumptions are systematically varied. During this step, the model’s contextual environmental factors will be held constant, allowing the team to explore the sensitivity and dependency of outcomes (patterns of mis-implementation) to changes in assumptions about agent behavior and characteristics. Leveraging computational power to build up a robust statistical portrait of model dynamics and parameters, where assumptions are systematically varied, will allow for appropriate interpretation of model behavior, including the relationships between individual, organizational, and external contextual factors.

##### Step 4: model analysis to generate insights by manipulating potential levers

In this step, the team will introduce changes in individual and organizational agent knowledge and behavior and in contextual variables to explore effects on mis-implementation. This will help point to modifiable individual and organizational agent characteristics that, if addressed, can reduce mis-implementation in a variety of external conditions (i.e., essential leverage points). The ABMs can provide not only aggregate outcomes, but also information about how the relative importance and effect of different factors may vary across contexts and state health departments.

## Discussion

Mis-implementation has not been studied nor have adequate measures been developed to fully assess its impact in the field of population-based cancer control and prevention. This study is timely and important because (1) cancer is highly preventable, yet existing evidence-based interventions are not being adequately applied despite their potential impact; (2) pilot research shows that mis-implementation in cancer control is widespread; and (3) ABM is a useful tool to more fully understand mis-implementation complexities and dynamics. Results from this study can help shape and inform how state health departments guide and implement effective cancer control programs as well as continue to test the utilization of agent-based modeling to inform chronic disease and cancer control research.

### Furthering the debate about terminology

As the multi-disciplinary field of implementation science has developed over the past 15 years, scholars from diverse professions have attempted to document the many overlapping and sometimes inconsistently defined terms [101]. Many important contributions to implementation science originate from non-health sectors (e.g., agriculture, marketing, communications, management), increasing the breadth of literature and terminology. To bridge these sectors, a common lexicon will assist in accelerating progress by facilitating comparisons of methods and findings, supporting methods’ development and communication across disciplinary areas, and identifying gaps in knowledge [102, 103].

When we began the pilot work for this project in 2014 [22], we cataloged a number of established, relevant terms related to our scope, including:

- De-implementation: “abandoning ineffective medical practices and mitigating the risks of untested practices [23]”;
- De-adoption: “rejection of a medical practice or health service found to be ineffective or harmful following a previous period of adoption… [104]”;
- Termination: “the deliberate conclusion or cessation of specific government functions, programs, policies or organizations [105]”;
- Overuse: “clinical care in the absence of a clear medical basis for use or when the benefit of therapy does not outweigh risks [106]”;
- Underuse: “the failure to deliver a health service that is likely to improve the quality or quantity of life, which is affordable [107]”;
- Misuse: typically synonymous with “medical errors [108]”

**Fig. 4:**
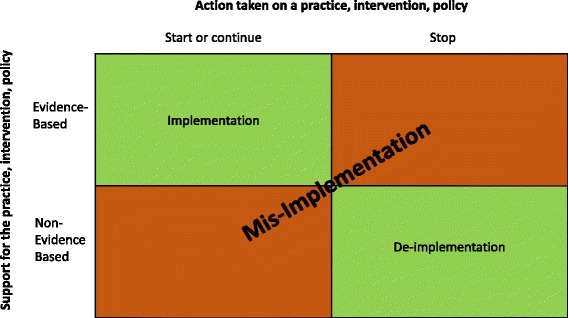
Conceptualization of the definition of mis-implementation

Because the goal of our project is to describe and model two related phenomena (ending of effective interventions or continuation of ineffective interventions), there was not an existing term that described the dual processes that we are addressing. Figure 4 depicts the bi-directional meaning of mis-implementation. The green cells show the desired implementation of effective programs and de-implementation of ineffective programs. The orange cells show the undesirable ending of effective programs and continuation of ineffective programs, which we label as mis-implementation. We welcome input on terminology as we learn more about these processes. We hypothesize that some of the underlying reasons and interaction of agents may differ for ending effective interventions versus continuing ineffective interventions. This will allow us to continue to refine terminology over time.

While planning the current project, we also realized that the use of the term mis-implementation may give some practitioners unease in that our study might reflect negatively on their day-to-day programs to decision-makers (e.g., public health leaders, policy makers), rather than identifying areas for improvement. These concerns have been addressed with a practice advisory group from the beginning of the project, and the team intends to frame the study goals carefully, including use of a project title and description that imparts positive, actionable outcomes: “Public Health in Appropriate Continuation of Tested Interventions” (Public Health in ACTION).

### Utility of agent-based modeling

A strength of the study is the use of ABMs in combination with quantitative and qualitative methods to provide insights about how and why mis-implementation occurs and how specific policies may affect mis-implementation. These ABMs will explicate the dynamic interaction between individual decision-makers operating within organizations and influenced by external factors. An important policy utility comes from using the models to capture how variation in existing individual and organizational factors may reduce mis-implementation. Experiments using the models will allow comparison of baseline outcomes to outcomes as individual and organizational factors are changed to represent potential intervention scenarios. Insights generated by the models can be used to design novel strategies and policies that have the potential to effectively reduce mis-implementation. As these strategies become evident, the team will frequently seek input from the advisory group in order to maximize the real-world utility of the findings.

Like all ABMs, the planned models will require the team to make certain stylized assumptions about real-world structures and processes, especially when relevant data are not available. Translating the data the team collects in the initial phases of the project into the models in order to characterize individual and organizational behavior will require us to identify the most appropriate factors for inclusion, and develop appropriate ways to quantify these in a computational framework [43]. Many elements included in the models—including those related to external support and opposition for programs, barriers to decision-making, organizational climate, and individual capacity—although theoretically justified, are inherently difficult to measure and quantify. Therefore, while the models will elucidate important dynamics involved in mis-implementation and identify key leverage points for improving decision-making, they are not intended to provide precise, quantitative forecasts of how organizational changes will affect implementation decisions. Additionally, this is the first known attempt to computationally model the process involved in the implementation and mis-implementation of programs within health departments. As such, future modeling may be warranted to further enhance our findings and apply the models to other specific contexts.

### Limitations

The study has a few limitations. While the team has plans to ensure the highest response rates [72, 73], high turn-over and workload demand of state health department workers may impede data collection efforts [109, 110]. Based on previous research and state-of-the-art methods [51, 52, 54, 72, 73], the team will take multiple steps to ensure a high response rate and will also compare respondents with non-respondents. There are limitations on how fully and accurately survey self-report responses can capture mis-implementation frequency and patterns across complex multi-faceted statewide programs.

### Summary

A richer understanding of mis-implementation will help us better allocate already limited resources more efficiently, especially among health departments where a significant portion of cancer control work is contracted or performed in the USA [22]. This knowledge will also allow researchers and practitioners to prevent the continuation of ineffective programs or discontinuation of effective programs [22]. The team anticipates that the study will result in replicable models that can significantly impact mis-implementation in cancer control and can be applied to other health areas.

## Abbreviations

ABM: Agent-based model(ing)
CDC: Centers for Disease Control and Prevention
EBDM: Evidence-based decision-making
NACDD: National Association of Chronic Disease Directors

## Footnotes

1 We use the term “programs” broadly to include a wide range of interventions from structured behavioral change strategies to broad policy approaches to cancer screening promotion initiatives.
